# Deviance detection by a P3-like response in rat posterior parietal cortex

**DOI:** 10.3389/fnint.2012.00127

**Published:** 2013-01-04

**Authors:** Allicia Imada, Allyn Morris, Michael C. Wiest

**Affiliations:** Neuroscience Program, Wellesley CollegeWellesley, MA, USA

**Keywords:** frontal cortex, P300, ERP, MMN, evoked potentials, auditory

## Abstract

To better understand sensory processing in frontal and parietal cortex of the rat, and to further assess the rat as a model of human frontal-parietal processing, we recorded local field potentials (LFPs) from microelectrode arrays implanted in medio-dorsal frontal, and posterior parietal cortex of awake rats as they were presented with a succession of frequent “standard” tones and infrequent “oddball” tones. Extending previous results from surface recordings we found, after controlling for the frequencies of the standard and oddball tones, that rat frontal and parietal-evoked LFPs (eLFPs) exhibit significantly larger N1 (~40 ms latency), P2 (~100 ms), N2 (~160 ms), P3E (~200–240 ms), and P3L (~300–500 ms) amplitudes after an oddball tone. These neural oddball effects could contribute to the automatic allocation of attention to rare stimuli. To determine whether these enhanced responses to rare stimuli could be accounted for in terms of stimulus-specific neural adaptation (SSA), we also recorded during single-tone control sessions involving frequent standard, or infrequent oddball beeps alone. We compared the difference between rare-tone and frequent-tone response amplitudes in the two-tone context (oddball effect) or single-tone context which isolates the contribution of SSA (SSA effect). An analysis of variance (ANOVA) revealed a significant main effect of tone context on rare-tone response enhancements, showing that the rare-tone enhancements were stronger in the two-tone context than the single-tone context. This difference between tone contexts was greatest at the early P3E peak (200–240 ms post-beep) in parietal cortex, suggesting true deviance detection by this evoked response component, which cannot be accounted for in terms of simple models of SSA.

## Introduction

Rare auditory stimuli presented to human subjects evoke larger amplitude peaks in the average electro-encephalogram (EEG) response, known as the event-related potential (ERP), than more frequently presented tones (Wronka et al., 2008). This is known as an “oddball effect.” Some of these ERP peaks are thought to reflect the automatic allocation of attention to salient stimuli (Picton and Hillyard, 1974; Linden, 2005; Polich, 2007). Because ERP responses are altered in multiple neurological disorders such as schizophrenia (Pfefferbaum et al., 1989), understanding their neural origin may provide more insight into pathological conditions as well as normal attentional processing. Moreover, passive paradigms like this “passive oddball” paradigm may be advantageous for clinical testing of infants or patients with dementia, who have limited ability to follow instructions for an active task.

An ERP typically exhibits characteristic peaks, termed N1, N2, P1, P2, and P3 for their latency and sign (N for negative and P for positive voltage peaks). Here we focus on the N1 (90 ms), P2 (170 ms), and P3 (~300 ms, a.k.a. P300), which have been reported to be modulated by attention in humans (Picton and Hillyard, 1974; Naatanen and Picton, 1987). The amplitude of P300 in particular is thought to reflect stimulus salience, phasic attentional shifts, and attentional resource allocation (Sutton et al., 1965, 1967; Isreal et al., 1980; Kramer and Strayer, 1988; Polich, 1989; Soltani and Knight, 2000). The human P3 has moreover been associated with cognitive processing such as comparisons in working memory (Polich, 2007) and even conscious perception (Del Cul et al., 2007). It is among the most reliably altered features of ERPs in neurological disorders (Pfefferbaum et al., 1989; Linden, 2005). For example, schizophrenic patients exhibited reduced P3 amplitudes in comparison to control subjects without the disorder (Pfefferbaum et al., 1989).

Auditory N1 (50–80 ms) and P2 (130 ms) peaks have been identified in rats which may offer viable models of their human counterparts, though some species differences have been noted (Knight et al., 1985). Similarly, a P3 component has been reported which is larger in response to rare than frequent tones in a passive oddball context (Yamaguchi et al., 1993; Shinba, 1997; Sambeth et al., 2003; Hattori et al., 2010), as recorded from the brain surface over frontal, and parietal cortex. As in human EEG recordings (Wronka et al., 2008), distinct so-called P3E, and P3L waves can be identified at different latencies. However, in the rat literature these two components have been reported alone in separate studies: the early component was found around 240 ms (Yamaguchi et al., 1993) but others measured a P3-like potential at 380 ms (Sambeth et al., 2003). This apparent discrepancy in the literature on rat P3 is one motivation for the current study measuring oddball effects in terms of depth local field potentials (LFPs) recorded in rat frontal and parietal cortex.

Another ERP component that is reported to signal novelty in humans is the mis-match negativity (MMN). This is a relative negativity in the oddball ERP relative to the standard that peaks between 150 and 250 ms post-stimulus (Naatanen et al., 2007). This ERP component can overlap in latency with the N2 peak, but the two have been studied as distinct components (Folstein and Van Petten, 2008). Similarly, the MMN can overlap with the N1 peak but is considered a distinct component (Campbell et al., 2007). The MMN is of particular interest in studies like ours where stimuli are passively delivered and no behavioral response is required from the subjects, because it is observed irrespective of whether voluntary attention is directed to the stimuli (Naatanen et al., 2007). Moreover, the MMN is of clinical importance as it predicts the outcome of coma patients (Kane et al., 1993) and is altered in autism (Ferri et al., 2003). If the MMN manifests in rats, they could represent a valuable model for dissecting its neural mechanisms. One recent study of auditory cortex in rats found an MMN-like novelty-sensitive component (Nakamura et al., 2011), while another did not (von der Behrens et al., 2009), so this apparent discrepancy in the literature is another motivation for our study of deviance detection in rat cortex.

We also aimed to better localize the cellular generators of surface-recorded ERPs in the brain. Beyond that, as noted above, a motivation for pursuing these effects in rodents is that while invasive recordings in humans are only rarely feasible, depth recordings in rats afford the possibility of unit recordings and cellular-level manipulations [such as optogenetic targeting of specific cell-types (Cardin et al., 2009)] that could reveal the mechanisms of the oddball effect—which are likely to also constitute mechanisms of automatic bottom-up attention.

In particular, a recent series of studies has examined the possibility that oddball effects observed in auditory cortex in rats and cats might be accounted for in terms of stimulus-specific adaptation (SSA) to repeated stimuli. SSA is manifested by auditory cortical neurons (Squires and Donchin, 1976; Condon and Weinberger, 1991; Szymanski et al., 2009) and thalamic neurons in rats (Anderson et al., 2009; Yu et al., 2009), but not by thalamic neurons in cats (Ulanovsky et al., 2004). In principle SSA could account for an enhanced response to rare-tones among more frequent standards, because neurons responding to the standard would be more fatigued relative to neurons responding to the oddball tone. On the other hand, “true deviance or novelty detection,” in which an enhanced response signals an implicit comparison between current and previous stimuli in a series, cannot be achieved by SSA alone (Farley et al., 2010; Taaseh et al., 2011). The reason is that if only adaptation is in effect (i.e., no facilitation), then adding a frequently repeated standard tone to a sequence of rare oddballs can only further fatigue the neurons that respond to the oddball tone, leading to *equal or smaller* oddball responses than would be expected in the absence of the standards. Recent reports conflict as to whether SSA among auditory cortex neurons can account for enhanced responses to deviant oddball tones: a group recording in cat auditory cortex concluded that SSA among neurons there could account for the oddball effect they observed (Ulanovsky et al., 2003), while two other studies concluded that SSA among auditory cortical neurons could not account for the deviance detection they observed in rats (Taaseh et al., 2011), or the deviance detection evidenced in human ERPs (Farley et al., 2010).

To address these issues, we recorded LFPs in two cortical areas downstream from auditory cortex, posterior parietal, and frontal cortex, while rats listened passively to sequences of rare oddball and frequent standard tones. We reason that any neural oddball effect that will be manifested psychophysically as an attentional enhancement, must be manifested neurally somewhere along the processing hierarchy, which culminates in parietal and frontal cortex where behavioral responses are generated. We found evoked peaks that were significantly enhanced in both frontal and parietal cortex in response to low-probability tones. To test whether these oddball amplifications could be accounted for in terms of SSA, we also recorded during equivalent single-tone sessions, and compared oddball effect sizes to SSA effect sizes measured in the absence of a background of standards.

## Materials and methods

### Subjects and stimuli

Fourteen male Long–Evans rats, *Rattus norvegicus* [500–700 g, Charles River Laboratories (Wilmington, MA)]. Rats were housed in pairs (before electrode array implantation surgery) or individually (after surgery) in the Wellesley College Animal Care Facility on a 12:12 light/dark schedule (lights on at 6 am/off at 6 pm). LFP recordings took place during the day in a standard operant chamber (80003NS, Lafayette Instrument) housed in an actively ventilated sound-attenuating outer chamber. The front wall of the standard operant chamber was covered with cardboard to prevent interaction with the lickometers and nose poke in the chamber. The Wellesley College Institutional Animal Care and Use Facility approved all methods.

In the *two-tone passive oddball paradigm*, rats listened to a series of pure tones generated by ABET-II software (Lafayette Instruments, Inc.) from a speaker mounted on the upper left side of the front wall of the operant chamber. There was no training involved but rats were not allowed to sleep and “drowsiness” as evidenced by large-amplitude 10-Hz rhythms visible in the LFP (Wiest and Nicolelis, 2003; Fontanini and Katz, 2005) was discouraged by occasionally opening the door of the chamber and/or offering a drink of water to the rat manually. In general rats rarely showed any overt behavioral response to the stimulus tones, and usually sat quietly with occasional exploratory episodes. The auditory stimuli consisted of a frequent standard tone (83.33% of trials), or a rare oddball tone (16.67% of trials), each lasting for 100 or 50 ms in different sessions. The different tone durations are not expected to qualitatively change the results because evoked potentials reflect only stimulus onset-responses for tones of short duration (Naatanen and Picton, 1987); therefore we combined the 100 and 50 ms sessions in our analysis. (Only 8 of 194 recording sessions were 50 ms sessions; all of these were in one animal.) The inter-tone interval (ITI) was either 1 or 2 s, depending on the session. Our dataset did not exhibit significant differences between the 1 and 2 s ITIs. For example, the mean P3E peak amplitude (defined below) was 10 SE 1 μV in 2 s sessions as compared to 11 SE 1 μV in 1 s sessions. For Hi-Odd sessions, the higher-pitched (3000 Hz, 80 dBA) tone served as the rare stimulus; for Lo-Odd sessions, the lower-pitched (1500 Hz, 72 dBA) tone served as the rare stimulus. Hi-Odd and Lo-Odd sessions were collected on the same or successive days to allow for direct comparison as described below under Data Analysis. When sessions were collected on the same day, we varied the order in which Hi-Odd and Lo-Odd sessions were recorded to account for the potential effects of long-lasting habituation in the second recorded session. For comparison with the two-tone oddball sessions, *single-tone oddball-only and standard-only sessions*, with the same timing of tones but omitting the standard or oddball beeps, respectively, were also collected in pairs on the same or successive days. The number of repetitions of oddball beeps in recorded single- and two-tone sessions with greater than 75 trials (oddballs + standards, see Data Analysis below) varied between a minimum of 23 and a maximum of 663, with a median of 113 (mean 188, SD 157), while the number of standard beeps varied from 174 to 2559 with a median of 557 (mean 634, SD 389).

### Multi-electrode array implantation surgery

For implantation surgery, rats were anesthetized with isofluorane (1–2% in O_2_), and placed in a stereotaxic apparatus. Chronic 32-microelectrode arrays (Innovative Neurophysiology, Inc.) were implanted in right frontal (2.0 mm anterior to bregma, 0.75 mm dextrolateral to the midline and 1.5 mm beneath the brain surface) and right parietal cortex (4.15 mm posterior to bregma, 3.5 mm dextrolateral to the midline and 1.2 mm beneath the brain surface). The frontal array was a 2 × 16 grid and the parietal array was a 4 × 8 grid, both with an inter-electrode spacing of 150 μm and row spacing of 300 μm. Ground wires from each array were attached to three skull-screws (making contact with brain surface at left frontal, left parietal, and right occipital locations), and arrays were fixed in place with dental cement. After surgery, rats were allowed to recover for 1 week before beginning recording. All rats were weighed and assessed daily for signs of pain for one week following surgery.

### Electrophysiological recordings

LFP activity was recorded from frontal and parietal cortex during tone detection sessions with a Cerebus Data Acquisition System (Blackrock Microsystems) at 1000 Hz sampling rate and bandpass filtered offline between 0.5 and 250 Hz. LFPs were referenced to ground (see above). LFPs were transferred via NeuroExplorer to Matlab for data analysis. Further details on surgery and recording procedures can be found in Wiest et al. (2007).

### Data analysis

#### Pre-processing

LFPs recorded using a Blackrock Neural Signal Processor were transferred to MATLAB via NeuroExplorer and analyzed using custom MATLAB routines. Because a comprehensive analysis of the topography of eLFP responses within frontal and parietal areas is beyond the scope of this study, and because neighboring LFPs tend to be highly redundant, we chose a single LFP each from frontal and parietal cortex to analyze for each session. The LFPs were chosen as the highest quality electrode in a session based on visual inspection of raw-evoked LFPs (eLFPs) before throwing out artifact trials as described next. LFPs segments from 100 ms before the stimulus onset to 500 ms after were extracted to define trials. Trials in which the signal exceeded ±1500 μV or contained a flat line were automatically discarded. Any other artifact trials that passed the cut were manually rejected. If fewer than 75 trials (oddballs + standards) in a session survived these cuts, then the session was omitted from the analysis. Linear trends were removed from each trial using detrend.m from the Chronux package. After pre-processing trials were averaged to obtain the ERP for each type of tone in each session. To remove potential biases due to baseline drift the mean was subtracted from each eLFP. The eLFPs were then averaged across sessions to obtain grand average eLFPs for each area, in the two-tone and single-tone contexts.

#### eLFP peak definitions

Because the latencies of eLFP peaks vary among animals, which could obscure systematic changes in their amplitudes, we also calculated eLFP peak amplitudes and latencies for each session from each animal separately, based on a set of time windows derived from the grand average eLFPs and consideration of the individual session eLFPs. We found the P2 peak to be the most robust of the four we considered, so our algorithm identified this peak first, as the highest peak between 48 and 140 ms after the beep onset. If the P2 peak was found on the late edge of the window, then the window was lengthened to 150 ms and the search was repeated. The N1 was then identified as the minimum between 30 and 100 ms post-beep, but constrained to be prior to the P2. The N2 was defined as the minimum voltage between the P2 and 300 ms. We defined an early P3E as the maximum between 140 and 300 ms, but constrained to be after the P2. Finally the late P3L was defined as the maximum between 300 and 500 ms after the tone onset.

#### Oddball and SSA effect calculations

The oddball effect size for each peak was calculated as the difference between the peak amplitude in response to the tone presented as an oddball vs. the amplitude in response to the *same* tone presented as a standard:

ΔODD=AMP(High-pitched oddball)−AMP(High-pitched standard).

This subtraction controls for the likelihood that different pitches elicit differing response amplitudes even when the different pitches are equally probable (Knight et al., 1985), and isolates the effect of stimulus probability, or rarity, on the response amplitude.

Likewise, we used single-tone recording sessions to isolate the effects of ITI on each peak amplitude, as the difference between response amplitude when the tone was presented rarely (in an Oddball-only session) vs. the response amplitude when the same tone was presented frequently (in a Standard-only session):

ΔSSA=AMP(High-pitched lone oddball)−AMP(High-pitched lone standard).

Average oddball effects and SSA effects were compared to zero using two-tailed paired *t*-tests with a significance criterion Bonferroni corrected for 10 comparisons (=2 areas × 5 peaks; *p* < 0.005). A three-factor analysis of variance (ANOVA) with factors AREA (levels = frontal, parietal), CONTEXT (levels = two-tone, single-tone), and PEAK (within-subjects, levels = N1, P2, N2, P3E, P3L) was used to assess whether the oddball effects were significantly larger than the SSA effects.

## Results

In order to study brain response enhancements to rare stimuli, LFPs were recorded in medio-dorsal frontal and posterior parietal cortex while awake rats were exposed to sequences of tones, in two-tone “oddball” sessions and separately in single-tone “SSA” sessions designed to isolate the effect of stimulus-specific neural adaptation (SSA). Frontal and parietal LFP recordings (counted separately) during two-tone oddball sessions comprised 61 session-pairs (high-pitched oddball and low-pitched oddball) in 14 rats (31 frontal session-pairs, 30 parietal session-pairs; the numbers are not identical due to two animals with data from only one area). Recordings during single-tone SSA sessions comprised 32 session-pairs (frequent single-tone and infrequent single-tone) in 9 rats (18 sessions-pairs each for frontal and parietal cortex).

**Figure 1:**
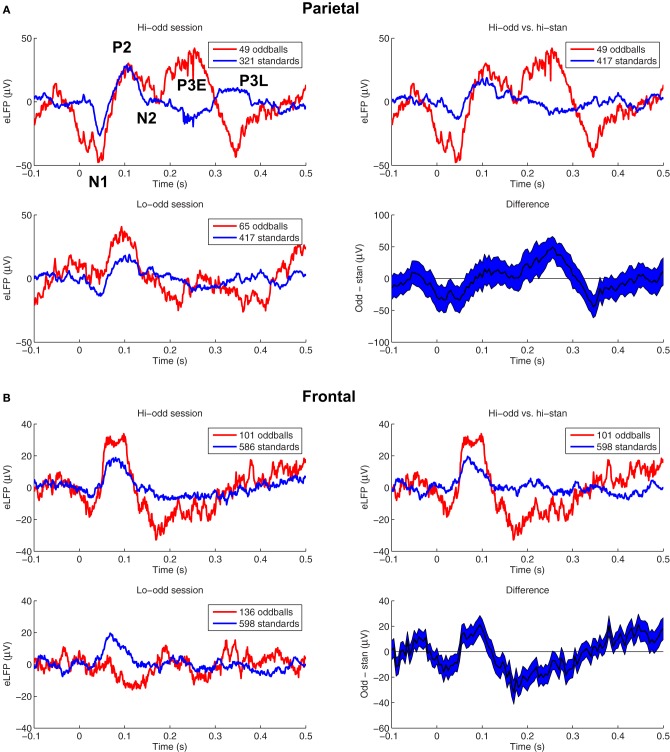
**Auditory eLFPs recorded during two example pairs of two-tone oddball sessions. (A)** *Top left panel*—eLFPs recorded in parietal cortex of one rat while the high-pitched tone was presented rarely as oddball (*red*) and the low-pitched tone was presented frequently as standard (*blue*). The five eLFP peaks that we studied are labeled: N1 (~40 ms), P2 (~100 ms), N2 (~160 ms), an early P3E (~250 ms), and a late P3L (>300 ms). *Bottom left panel*—eLFPs recorded from the same frontal electrode in the same rat as in the top left panel, in a separate session with pitches reversed: the high-pitched tone was presented as standard (*blue*) and the low-pitched tone was presented as oddball (*red*). *Top right panel*—Comparing oddball and standard response amplitudes from a single two-tone session confounds frequency-dependent responding with any potential enhanced responses to rare stimuli. Therefore, we compare responses to the high-pitched tone presented as oddball (*red*), with responses to the same high-pitched tone presented as standard (*blue*) in a matched session in the same animal. *Bottom right panel*—shows the difference between the oddball and standard eLFPs from the top right panel, with a 68% confidence interval based on the variability across trials. The positive difference around 250 ms post-stimulus corresponds to an enhanced parietal P3E amplitude in response to the rare oddball tone. **(B)** Shows eLFPs recorded in frontal cortex of a different rat than **(A)**, during a high-pitched oddball session (*top left panel of* ***B***), a low-pitched oddball session (*bottom left panel*), a comparison of the high-pitched oddball eLFP with the high-pitched standard eLFP (*top right panel of* ***B***), and the difference curve with 68% confidence interval (*bottom right panel*). In this pair of sessions the rare-tone response was amplified at the N1, P2, N2, and P3L peaks.

We first wanted to assess the extent to which the responses to rare-tones were enhanced at our frontal and parietal recording sites. Figure 1 shows eLFPs from two example pairs of sessions in different rats. The peaks labeled in the top left panel of Figure 1A define our nomenclature for the components of the eLFPs we studied (see “Materials and Methods”): N1 at a latency of about 40 ms, P2 near 100 ms, and an “early” P3E around 200–250 ms. We also examined an N2 component between the P2 and P3E peaks, although in this session the minimum potentials in that time window were barely negative. We also considered a “late” P3L peak after 300 ms latency, which in this session appeared at different latencies in the two-tone conditions (oddball and standard). The example in Figure 1A shows eLFPs recorded in parietal cortex. In the pair of sessions illustrated in Figure 1A, the response to the rare oddball stimulus was larger in amplitude than the response to the frequent standard tone, regardless of whether the oddball was the higher-pitched (top left of Figure 1A) or the lower-pitched tone (bottom left of Figure 1A). Nevertheless, since we are interested in the differential response to rare and frequent tones, rather than different obligatory responses to different pitches, the meaningful comparison is between the high-pitch oddball eLFP and the high-pitch standard eLFP (top right of Figure 1A). In this example pair of sessions, the rare-tone response enhancement is strongest at the early P3E peak.

Figure 1B shows frontal eLFPs recorded in a pair of sessions in a different rat. In this case the response to the low-tone presented as a rare oddball was smaller overall than the response to the high-tone presented as standard (bottom left panel), highlighting the need for our subtraction procedure (right panels) to avoid confounding pitch-related response differences with response differences based on stimulus probability.

**Figure 2:**
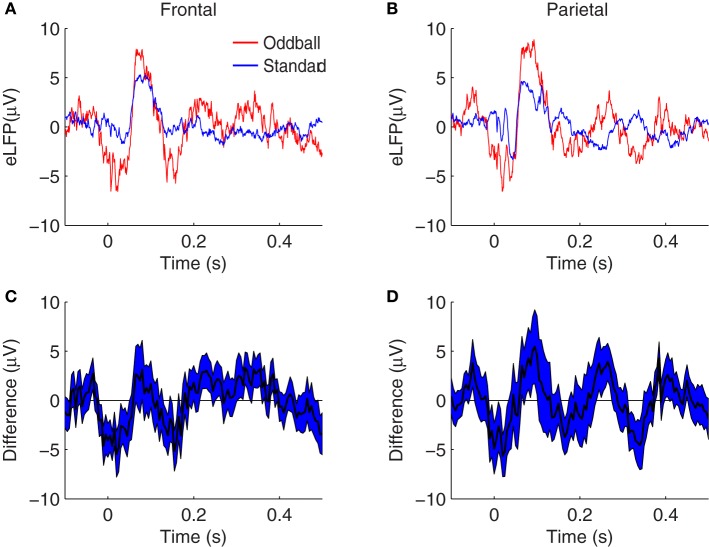
**Grand average eLFPs for the two-tone oddball paradigm. (A,B)** eLFPs averaged over session-pairs recorded in14 rats, from frontal **(A)** and parietal **(B)** cortex, for oddball tones (*red*) and standard tones (*blue*) separately. **(C,D)** plot the grand average difference between the oddball and standard eLFPs at frontal **(C)** and parietal **(D)** electrodes, with the shaded region representing a 68% confidence interval based on the variability across sessions. In both brain regions, evoked responses tend to be larger in amplitude when the tone is presented as a rare oddball, as opposed to when the same tone is presented as a frequent standard.

Our first approach to the question of whether the N1, P2, N2, P3E, and P3L peaks manifest larger amplitudes in response to rare oddballs in a background of frequent standards was by means of grand average differences between oddball and standard eLFPs (Figure 2). The trend in both frontal and parietal cortex is toward larger amplitude peaks in response to the tone presented as rare oddball, at each of the components we focused on. However, Figure 2 also suggests the possibility that peaks may be shifted with respect to one another in the oddball and standard eLFPs. For example, the positive peaks after 150 ms appear at different latencies in the oddball and standard eLFPs (Figures 2A,B). Thus, the raw difference between oddball and standard eLFPs may not be the most appropriate way to assess a putative enhancement of a given peak. Moreover, because eLFP peaks appear at different latencies in different animals, and even in different sessions recorded from the same animal, significant effects could be obscured in the grand average eLFP difference curves.

We therefore calculated N1, P2, N2, P3E, and P3L peak amplitudes for each session, and calculated oddball effects based on the set of peak amplitudes rather than the difference curves. While the differences curves can only compare identical latencies between oddball and standard conditions, the peak-based analysis compares corresponding peak amplitudes in the two conditions.

**Figure 3:**
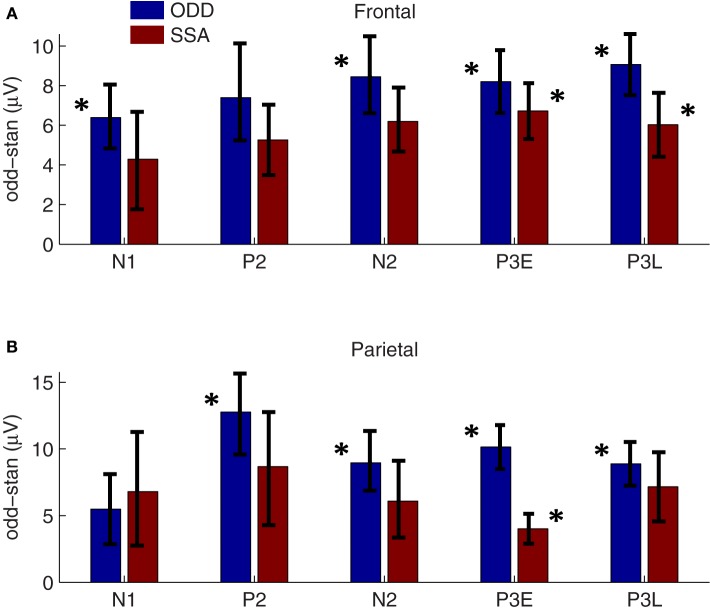
**Oddball and SSA effect sizes.** *Blue bars*: Average oddball effect sizes (across 61 two-tone session-pairs in 14 rats) are plotted for each eLFP peak in frontal **(A)** and parietal **(B)** cortex. A positive effect size corresponds to a larger response to the rare-tone (for both positive and negative eLFP peaks). Asterisks flanking the blue bars denote significant oddball enhancements based on Bonferroni-corrected two-tailed *t*-tests (*p* < 0.005). From left to right the oddball effect *p*-values are **(A)**: 0.0003, 0.007, 0.0001, 0.00001, 0.000002; **(B)**: 0.046, 0.0002, 0.0005, 0.000001, 0.000008. Error bars denote standard error. *Red bars:* SSA effect sizes averaged over 36 session-pairs from 9 rats are plotted alongside corresponding oddball effect sizes at frontal electrodes **(A)** and parietal electrodes **(B)**. Asterisks flanking the red bars denote significant oddball enhancements based on Bonferroni-corrected two-tailed *t*-tests (*p* < 0.005). From left to right the SSA effect *p*-values are **(A)**: 0.1, 0.009, 0.1, 0.0002, 0.002; **(B)**: 0.1, 0.05, 0.1, 0.002, 0.01. Error bars denote standard error. A three-way ANOVA revealed a significant main effect of tone context on rare-tone response enhancements (*dof* = 484, *F* = 7.9, *p* = 0.019), showing that the response enhancements cannot be accounted for by SSA alone.

Oddball effect sizes were calculated as the difference between the response amplitude to a tone presented as the oddball and the response amplitude to the same tone presented as the standard, for the positive peaks, and the reverse for the negative peaks. Thus, enhanced peak amplitude responses to the rare-tone correspond to positive effect sizes for both the positive and negative peaks (Figure 3, blue bars). The mean peak amplitudes along with their standard errors across sessions are shown in Table 1. We observed oddball enhancements in both frontal and parietal cortex of all five eLFP peaks in response to rare-tones as compared to frequent tones. All of these were statistically significant by our Bonferroni corrected criterion (two-tailed *t*-tests, *p* < 0.005) except the frontal P2 enhancement (*p* = 0.007) and the parietal N1 enhancement (*p* = 0.046). These enhanced responses cannot be due to different obligatory responses to different pitches, because our procedure compares responses to the same pitch when it is presented as an oddball or a standard in different (paired) sessions. Thus, these enhancements could contribute to the involuntary neural allocation of attention to rare stimuli.

**Table 1:** **Mean eLFP peak amplitudes in microvolts for rare (oddball) and frequent (standard) tones presented in two-pitch sessions, in frontal and parietal cortex**.

**Peak**	**Frontal oddball**	**Frontal standard**	**Parietal oddball**	**Parietal standard**
N1	12 SE 2	5.6 SE 0.9	13 SE 3	8 SE 1
P2	19 SE 3	11 SE 1	24 SE 4	11 SE 2
N2	17 SE 2	8.6 SE 0.8	18 SE 2	9 SE 1
P3E	16 SE 2	7.5 SE 1	19 SE 2	8 SE 1
P3L	16 SE 2	6.9 SE 0.6	17 SE 2	8 SE 0.7

Standard errors (SE) are based on variability across sessions. One significant figure of the SE is shown; amplitudes are rounded in accordance with the SE.

**Figure 4:**
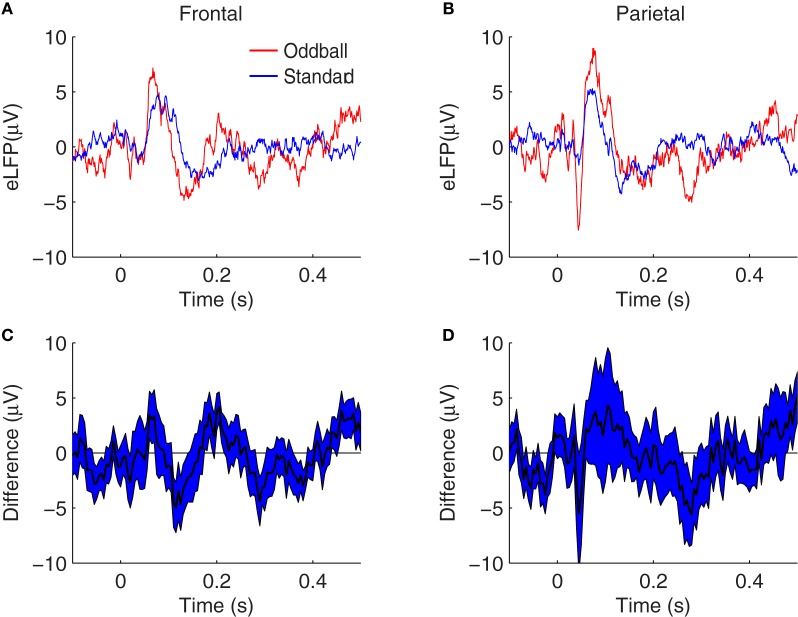
**Grand average eLFPs for the single-tone paradigm. (A,B)** eLFPs averaged over 36 session-pairs in nine rats, in frontal **(A)** and parietal **(B)** cortex, for tones presented at oddball repetition rate (*red*) or tones presented at standard repetition rate (*blue*) separately. **(C,D)** plot the grand average difference between the single-tone rare (oddball) and frequent (standard) eLFPs at frontal **(C)** and parietal **(D)** electrodes, with the shaded region representing a 68% confidence interval based on the variability across sessions. In both brain regions, evoked responses tend to be larger in amplitude when the tone is presented infrequently, as opposed to when the same tone is presented frequently: in this single-tone context the enhancement may be attributed to SSA. Axes are identical to those in Figure 2 to facilitate comparison.

To test whether these augmented response amplitudes could be accounted for by SSA, we compared these oddball effects to analogous effects calculated from pairs of single-tone recording sessions. In the single-tone sessions the background tones of a different pitch are omitted, so that only adaptation specific to the presented tone is in effect (i.e., SSA). In paired sessions, the single-tone is presented at standard repetition rates and separately at oddball repetition rates. Therefore, the difference between the response amplitude to the tone in the two contexts can be attributed to the different ITIs in the two paired single-tone sessions—but not to any putative memory comparison of the current stimulus with a recent history of different pitches, such as might occur in the two-tone context. Grand average single-tone eLFPs are shown in Figure 4. The same N1, P2, N2, P3E, and P3L peaks are identifiable in the single-tone eLFPs as in the two-tone data shown in Figure 2. Similar trends toward greater peak amplitudes in response to the rarer tone are evident in the single-tone responses as in the two-tone responses, except that the frontal N1 amplification and the parietal N2 effect are not visible in the raw eLFP differences (Figures 4C,D).

As for the two-tone eLFPs, averaging across sessions and subtracting the rare- and frequent-tone eLFPs directly could obscure significant effects with varying latencies, so as in that case we defined peak amplitudes as voltage maxima (or minima) in distinct latency windows for each eLFP and compared the peak amplitudes to assess potential effects of SSA. The mean peak amplitudes from the single-tone SSA data are shown in Table 2. Our primary question was whether oddball effects are larger than their corresponding SSA effects, which would indicate that SSA was not the only cellular mechanism contributing to the enhanced oddball responses. Figure 3 shows the average SSA effect sizes plotted (as red bars) alongside the oddball effects (blue bars). In the single-tone data the only statistically significant rare-tone enhancements (by our Bonferroni corrected criterion) occurred at the P3E peak in both parietal and frontal cortex, and at the P3L peak in frontal cortex. At every eLFP peak except for the parietal N1 the trend is toward larger response enhancements in the two-tone oddball context as compared to the single-tone pure SSA context.

**Table 2:** **Mean eLFP peak amplitudes in microvolts for rare (odd-only) and frequent (stan-only) tones presented in single-pitch sessions, in frontal and parietal cortex**.

**Peak**	**Frontal odd-only**	**Frontal stan-only**	**Parietal odd-only**	**Parietal stan-only**
N1	9 SE 3	4 SE 1	13 SE 5	6 SE 2
P2	14 SE 2	9 SE 1	19 SE 4	10 SE 2
N2	14 SE 2	8 SE 1	15 SE 3	9 SE 2
P3E	12 SE 2	5.1 SE 0.8	12 SE 0.8	8 SE 1
P3L	13 SE 2	6.8 SE 0.9	17 SE 3	8 SE 1

Standard errors (SE) are based on variability across sessions. One significant figure of the SE is shown; amplitudes are rounded in accordance with the SE.

To make a quantitative statistical comparison of the oddball effects with corresponding pure SSA effects, without incurring multiple comparisons, we applied a three-way ANOVA. The first factor was brain AREA, with frontal and parietal levels; the second factor was CONTEXT, with two-tone oddball and single-tone SSA levels; and the third factor was PEAK with within-subjects levels N1, P2, N2, P3E, and P3L. We observed a significant main effect of context (*dof* = 484, *F* = 7.9, *p* = 0.019), showing that the oddball effect is significantly larger than the SSA effect. The main effect of brain area was not significant (*p* = 0.07). There was a significant main effect of peak (*p* = 10^−9^), but we did not pursue *post-hoc* comparisons as we are interested in differences between the oddball and SSA contexts. These results demonstrate an overall greater rare-tone enhancement in the two-tone context, but do not localize that difference to frontal or parietal cortex, or to a particular latency window. Because the trend of the oddball vs. SSA difference is in the same direction in frontal and parietal cortex and at most of the peaks, the AREA × CONTEXT interaction effect was not significant (*p* = 0.1), and neither was the interaction between peak and context (*p* = 0.7). Nevertheless, we can note that the largest difference between oddball and SSA effects occurs at the P3E peak in parietal cortex (Figure 3).

To examine the possibility that our effect sizes might have been diminished in longer sessions due to increased adaptation of responses, we also ran our statistical analysis only considering the first 100 oddballs and the first 600 standards in sessions with greater numbers of stimuli. eLFPs and peak amplitudes were qualitatively similar (not shown), but the main effect of context was more highly significant (*dof* = 484, *F* = 17.4, *p* = 0.002), further supporting that the rare-tone enhancements we observed are stronger in the two-tone oddball context. There was also a significant main effect of brain area (*F* = 16.4, *p* = 0.002), showing that rare-tone enhancements were stronger in parietal cortex. Moreover, in this reduced data set the interaction between area and context was significant (*F* = 23.7, *p* = 0.0007), meaning that the difference between the two-tone and single-tone contexts was largest in parietal cortex. There was again a significant main effect of peak (*F* = 46.4, *p* < 10^−9^), but interactions between peak and the other factors were not significant. Again the largest difference between the two-tone and single-tone contexts occurred at the P3E peak in parietal cortex.

These results are consistent with the oddball effects and SSA effects visible in the grand average difference eLFPs (Figures 2, 4), where trends toward augmented responses are visible on the rare-tone eLFPs in both frontal and parietal cortex in the two-tone context, but there is little rare-tone enhancement visible in the parietal eLFPs in the one-tone context, particularly at the P3E peak.

## Discussion

To constrain the mechanisms that enhance evoked responses to infrequent auditory stimuli, we recorded medial-dorsal frontal and posterior parietal LFPs in unrestrained awake rats passively listening to frequent and infrequent tones presented in a two-tone oddball or single-tone pure SSA context. To control for potentially different obligatory responses to the different pitches, low-frequency oddball, and high-frequency oddball sessions were collected in pairs in varying order on the same or successive days, so that we only make comparisons between responses to the same pitch, presented in different contexts. Thus, the enhancements we measured could reflect automatic bottom-up allocation of attention to rare stimuli.

### Oddball response enhancements in frontal and parietal eLFPs

From the two-tone oddball session data we found significant “oddball effects,” representing larger responses to rarer tones, on N2, P3E, and P3L eLFP peaks in both frontal and parietal cortex. These results are consistent with previous surface recordings (Yamaguchi et al., 1993), although in another EEG study in rats the oddball enhancements did not reach statistical significance during their passive paradigm (Shinba, 1997). An enhancement of the N1 peak was significant in frontal cortex and an enhancement of the P2 peak was significant in parietal cortex. LFP recordings in anterior cingulate cortex of rats, about 1 mm deeper than our frontal recordings, showed a significant oddball enhancement of only the P2 peak (Hattori et al., 2010). However, the focus of the latter two studies was a comparison between passive and active oddball recordings, and they did not control for the pitch of the oddball and standard tones in the passive oddball experiments. As such, our findings extend previous results and appear to validate our approach focusing on peak amplitude measurements rather than direct subtraction of eLFPs in different conditions. The significant effects we observed on both early and late P3 peaks corroborates the existence of the two peaks in rats, but it remains unclear why only one or the other peak was measured in previous studies (early in Yamaguchi et al., 1993; Shinba, 1997; Hattori et al., 2010; late in Sambeth et al., 2003). Possibly latency variations among rats obscured effects in their grand average ERPs. The individual-rat ERPs presented by Yamaguchi et al. (1993) in their Figure 1 support that the late component is variable among animals.

In general our eLFP amplitudes tended to be smaller than similar measurements made by other groups (Yamaguchi et al., 1993; Sambeth et al., 2003). For example, our session-averaged peak amplitudes were all smaller than 25 μV, whereas others reported N1 amplitudes of 80–90 μV and P3 amplitudes on the order of 50–60 μV. Possible reasons for these differences include differences in auditory responses in the different areas sampled, differences in the intensity of the auditory stimuli, differences in the number of stimuli delivered per session, or differences in ITIs. The fact that the difference between two-tone and single-tone contexts became more statistically significant when we limited the number of tones analyzed in each session somewhat supports the possibility that large number of stimuli in some of our sessions led to more adapted responses than in the studies mentioned above. However, the raw amplitudes of eLFPs were similar whether we used all beeps from each session or only the first 100 oddballs and 600 standards. The most likely explanation for our smaller response amplitudes appears to be that our ITIs (1 or 2 s) were smaller than those used in the above studies. In fact one of those earlier studies noted that their response “amplitudes habituated a short inter-stimulus intervals.”

### Deviance detection by the early P3-like component in parietal cortex

The rare-tone enhancements we observed were significantly larger in the two-tone oddball context as compared to the single-tone SSA context. Therefore, the enhancement in response to rare-tones cannot be accounted for solely by SSA among the responding neurons between the periphery and the frontal or parietal recording sites, and may be considered as “true deviance detection” reflecting an implicit comparison of the current stimulus to the context of recent past stimuli. Every peak except the parietal N1 supported this trend, suggesting that the non-SSA mechanism responsible for the augmented rare-tone response in the two-tone context may be distributed across latencies and between frontal and parietal cortex. Alternatively it is possible that the extra rare-tone enhancement in the two-tone context may be inherited by frontal cortex from parietal cortex.

In either case, the deviance detection effect was dominated by the P3E peak in parietal cortex, where the difference between two-tone and single-tone contexts was greatest (Figure 3).

The effects we observed in parietal (and frontal) cortex should reflect response properties of neurons in upstream areas up to and including the recording sites. Adaptation of sub-cortical responses to repeated stimuli has been reported in the auditory thalamus (Anderson et al., 2009; Yu et al., 2009). Sub-cortical SSA has relatively broad specificity and requires short gaps between stimuli or constant stimulus, while cortical SSA has high specificity, long sensory memory, and relatively long latency. [Ulanovsky et al. (2003) reported an absence of adaptation in the auditory thalamus of cats.]

At the level of the cortex, studies differ as to whether SSA can account for the observed auditory oddball enhancements. Unit recordings in primary auditory cortex (A1) of awake cats exhibited SSA that did account for their enhanced responses to rare sounds (Ulanovsky et al., 2003, 2004), and the authors proposed SSA as a mechanism of sensory memory and novelty detection. In contrast, a study of multi-unit and LFP activity in auditory cortex of anesthetized rats concluded that SSA alone could not account for the oddball response enhancements they observed, which as such manifested “true deviance detection” (Taaseh et al., 2011). Similarly, another recent study of SSA among auditory cortical units, in awake rats, concluded that the adaptation properties of A1 units did not reflect “sensory novelty” in the same ways as the MMN of human ERPs (Farley et al., 2010), which appears variably in the N1 to N2 latency range (Naatanen et al., 2007). While it may be pointless to debate the semantics of true and ersatz “deviance detection,” the substantive disagreement among the studies is about whether SSA can account for the data at the level of A1 neurons and LFPs.

Our results show that the LFP response in posterior parietal cortex and medial frontal cortex exhibits novelty detection of a sort that cannot be accounted for solely by SSA, particularly in parietal cortex at a latency of 200–240 ms after the onset of a beep. This response could reflect synaptic inputs to parietal neurons, in the form of dendritic potentials, but might also include a component due to population spiking activity in the area. Thus, our results are consistent with the possibility of non-SSA novelty detection inherited from A1, but cannot rule out the possibility that the additional non-SSA enhancement is generated downstream of A1.

### The rat as a model of human ERPs

The most robust and studied novelty-related enhancements in the human ERP literature occur at the human P3 (Linden, 2005) and the MMN (Naatanen et al., 2007; Farley et al., 2010). The rat N1 and N2 that we studied here might represent rat analogs of the human MMN, in that they show enhanced responses to rare-tones; however, it is not clear that the enhancements we measured were inconsistent with SSA as a mechanism. On the other hand novelty-related amplification of the P3-like peak around 200–240 ms after the tone cannot be attributed entirely to SSA—as such it might rely on similar mechanisms as the human P3. The human P3 gets contributions from generators in multiple areas, but novelty-related enhancements are associated with an early frontal component (Linden, 2005). In this respect our result might point to species differences between rats and humans, since the oddball amplification we measured at the P3E peak was strongest in parietal rather than frontal cortex. Taken together our results extend the correspondence between frontal-parietal sensory processing in rats and humans (Sambeth et al., 2003), and begin to identify species differences. Combined unit and LFP recordings (Taaseh et al., 2011), or application of optogenetic manipulations (Cardin et al., 2009), in rodents could thus further elucidate the cellular basis of novelty detection in humans.

### Conflict of interest statement

The authors declare that the research was conducted in the absence of any commercial or financial relationships that could be construed as a potential conflict of interest.
